# Patient and Technology Selection for Focal Therapy in Prostate Cancer

**DOI:** 10.3390/cancers18132070

**Published:** 2026-06-25

**Authors:** Mustafa Dinckal, Rodrigo Rodrigues Pessoa, Julio Pow-Sang, Alice Yu

**Affiliations:** 1 Department of Urology, Kars Harakani State Hospital, Kars 36200, Turkey; 2 Department of Genitourinary Oncology, Moffitt Cancer Center, Tampa, FL 33612, USA

**Keywords:** prostate cancer, focal therapy, prostate biopsy, multiparametric MRI, PSMA PET scan

## Abstract

Focal therapy is emerging as an organ-preserving strategy for selected patients with localized prostate cancer, aiming to preserve urinary and sexual function while maintaining acceptable cancer control. However, patient and technology selection remain complex because prostate cancer is often multifocal, clinically significant lesions may be missed by imaging or biopsy, and long-term comparative oncological data are limited. This narrative review summarizes current evidence and consensus recommendations on oncological suitability, histopathological risk features, tumor burden, imaging assessment, anatomical considerations, functional priorities, and follow-up. We also discuss the complementary roles of multiparametric magnetic resonance imaging, prostate-specific membrane antigen positron emission tomography, micro-ultrasound, and artificial intelligence-assisted planning. Finally, we review how tumor location and proximity to critical structures guide selection among high-intensity focused ultrasound, cryotherapy, irreversible electroporation, transurethral ultrasound ablation, laser ablation, and photodynamic therapy. Focal therapy remains promising but requires careful selection, shared decision-making, and structured follow-up.

## Simple Summary

Prostate cancer is commonly treated with surgery or radiation, which can be effective but may also cause urinary incontinence, erection problems, and other quality-of-life concerns. Focal therapy aims to treat only the cancer-containing part of the prostate while preserving as much normal tissue as possible. However, this approach is suitable only for carefully selected patients, because prostate cancer can be multifocal and some cancer areas may be missed by imaging or biopsy. This review explains how clinicians can evaluate tumor features, imaging findings, patient priorities, and available treatment technologies when considering focal therapy. It also highlights the limits of current evidence, especially the lack of long-term comparative results. By summarizing current knowledge and uncertainties, this review may help clinicians make balanced, patient-centered decisions and guide future research.

## 1. Introduction

Prostate cancer is one of the most commonly diagnosed cancers in men worldwide, and its diagnosis and management processes have undergone significant changes over the past twenty years [1,2]. Although traditional radical treatment options such as surgery and radiation therapy offer high cure rates, they can lead to complications, such as urinary incontinence and erectile dysfunction, which seriously affect patients’ quality of life (QoL) [3]. Focal therapy (FT) has emerged as an organ-preserving alternative to radical treatments for intermediate-risk patients, with the aim of minimizing these morbidities [4].

The biological rationale for FT is based on the “index lesion” concept, which proposes that the dominant and most aggressive tumor focus may be the principal driver of disease progression and metastatic potential [5]. FT aims to selectively ablate the cancerous focus defined by imaging, along with its surrounding safety margin, while preserving the remaining healthy prostate tissue and critical anatomical structures [6,7]. However, this concept should be regarded as a clinically useful but still debated framework rather than an absolute biological rule. Prostate cancer is frequently multifocal, and clinically significant satellite lesions may be MRI-invisible or missed by biopsy. Consequently, focal therapy carries an inherent risk of undertreating occult out-of-field disease, and this uncertainty remains one of the major limitations of focal approaches [8].

The historical development of FT began in the early 1990s with hemigland cryotherapy and evolved into a precise treatment paradigm in the 2010s with the introduction of multiparametric MRI (mpMRI) and image-guided fusion biopsies [9]. Today, various energy sources, such as cryosurgery, high-intensity focused ultrasound (HIFU), irreversible electroporation (IRE), focal laser ablation (FLA), and photodynamic therapy (PDT) and transurethral ultrasound ablation (TULSA) are used for FT [10].

The success of FT critically depends on accurate patient selection. Despite advances in diagnostic tools, the multifocality of prostate cancer and the fact that mpMRI can miss 20% to 45% of clinically significant lesions remain major challenges [11]. Current consensus documents and international guidelines recommend that FT be administered, ideally within the framework of clinical trials or prospective registries, particularly in selected patients with moderate risk (ISUP Grade Groups 2 and 3), organ-confined disease, and a life expectancy of >10 years [10,12,13].

Importantly, the current evidence base for primary focal therapy should be interpreted with caution. Most available data derive from prospective or retrospective single-arm cohorts, registry studies, or studies comparing different focal therapy modalities, whereas randomized evidence directly comparing focal therapy with standard-of-care treatments such as radical prostatectomy or radiotherapy remains very limited. The only phase III randomized evidence in this field compared vascular-targeted photodynamic therapy with active surveillance in men with low-risk prostate cancer rather than with radical whole-gland treatment [14]. Therefore, focal therapy should currently be regarded as an investigational or selectively applied organ-preserving strategy rather than an oncologically equivalent alternative to established curative treatments. This limitation is particularly relevant in prostate cancer, where clinically meaningful differences in metastasis-free survival, cancer-specific survival, and overall survival may require follow-up exceeding 10–15 years.

The primary objective of this review is to identify the key criteria for patient selection for FT to achieve optimal oncological and functional outcomes and to evaluate how technological advances have shaped this process in light of contemporary evidence.

## 2. Patient Selection—Oncologic Considerations

### 2.1. Tumor Characteristics

From an oncological perspective, FT is based on the principle of eliminating the “index lesion,” which is expected to drive clinical outcomes despite the multifocal nature of prostate cancer. In this approach, tumor characteristics have a significant impact on treatment success and energy selection.

### 2.2. Tumor Volume

Tumor volume is one of the most critical factors determining technical success and recurrence risk. Lesions smaller than 1.5 cm or less than 20% of the prostate volume are generally considered ideal for FT. However, depending on proximity to vital structures, tumors up to 3 cm or 25% of the prostate volume can also be treated [9,15].

For successful oncological control, a safety margin of 5–10 mm should be added around the visible lesion [12]. As the tumor grows, maintaining this margin becomes challenging and it may be necessary to move from a strict focal ablation to a broader quadrant or hemiablation strategies, which can increase the risk of surrounding tissue damage [6].

### 2.3. Histopathological Features

Histopathological features in patient selection are the most critical cornerstone determining the oncological success of FT. Understanding biological behavior of the disease determines which patients are suitable for an organ-preserving approach and which require radical treatment.

Grade Group 2 (Gleason 3 + 4): Patients with Grade Group 2 disease represent the most appropriate candidate subgroup for focal therapy, as they may benefit from active treatment while potentially avoiding the morbidity associated with radical whole-gland approaches [4,16].

Grade Group 3 (Gleason 4 + 3): FT may be offered in selected cases; however, patients must be warned that they have a higher risk of recurrence and require closer follow-up [12].

Cribriform Pattern: It is now classified as Gleason pattern 4 (aggressive) [17]. Its presence is directly associated with an increased risk of biochemical recurrence, distant metastasis, and cancer-related death [17]. A noteworthy point in the FALCON consensus is the view that FT should be preferred over AS in Grade Group 2 disease (even with pattern 4 ratio < 10%) if cribriform structure is present given that its presence increases the risk of rapid progression among these patients [9,12,18].

Intraductal Carcinoma (IDC-P): IDC-P is usually associated with genomic instability and high-grade invasive cancers [18]. The presence of IDC-P may indicate the presence of higher tumor volume and increased likelihood of pelvic lymph node metastasis [17]. These features may translate into MRI-invisible micrometastatic foci or occult extracapsular extension [17]. Therefore, these patients are at a substantially higher risk of failure following FT, including both in-field and out-of-field recurrences.

## 3. Patient Selection—Imaging Characteristics

Imaging methods play an indispensable role in patient selection and treatment planning for FT [19]. While multiparametric MRI (mpMRI) is currently the cornerstone of this process, new technologies such as PSMA PET and micro-ultrasound are emerging as complementary tools [20,21,22]. mpMRI is the gold standard for detecting clinically significant prostate cancer (csPCa) and determining the location of the index lesion [22,23]. mpMRI accurately determines the number, size, and location of tumors, thereby identifying whether the patient may be suitable for FT (e.g., exclusion of extracapsular extension—ECE) [12]. mpMRI provides a systematic framework for planning FT. In this process, signals from all mpMRI sequences (T2W, DWI, DCE) are combined to define the Imaging Lesion Zone (ILZ) [24]. An Ablation Zone (AZ) is created in this area by adding a 5–10 mm safety margin to compensate for the tendency of MRI to underestimate tumor size [4,24]. mpMRI clarifies the distance between structures such as the rectum, urethral sphincter, and nerve bundles and the tumor, enabling the decision on which energy source (HIFU, IRE, etc.) to use to reduce the risk of complications [7,24].

PSMA PET/CT is a tool that supports MRI, particularly in focal treatment planning, and improves risk classification. PSMA PET can detect lesions that are not visible on mpMRI but may be clinically significant in up to 29% of patients [25]. This minimizes the risk of selection error, with residual cancer remaining outside the treatment field after FT. Although the FALCON consensus states that FT should not be offered when MRI is unavailable or of poor quality, whether PSMA PET can replace MRI in patients with negative MRI but positive biopsy results remains a matter of debate [4]. Nevertheless, emerging evidence, including recent studies such as the PRIMARY II trial, suggests that PSMA PET may aid in excluding clinically significant prostate cancer and reducing the need for biopsy in patients with negative or equivocal mpMRI findings [26]. Recent evidence further supports the complementary role of PSMA PET/CT in local staging and treatment planning. Soeterik et al. evaluated 550 patients undergoing radical prostatectomy and compared MRI, PSMA PET/CT, and their combination for detecting extracapsular extension and seminal vesicle invasion. Although PSMA PET/CT alone had lower sensitivity than MRI for extracapsular extension, combining PSMA PET/CT with MRI increased sensitivity and negative predictive value for extracapsular extension and improved sensitivity, negative predictive value, and area under the curve for seminal vesicle invasion compared with MRI alone [27]. These findings support the use of PSMA PET/CT as a complementary rather than substitutive modality to mpMRI in focal therapy planning, particularly when accurate exclusion of occult extracapsular extension or seminal vesicle invasion is critical. In addition to lesion detection and local staging, PSMA PET-derived molecular risk scores may further refine patient selection in the future. Karpinski et al. recently proposed PPP3, an international PSMA PET-based risk classification integrating PROMISE-derived molecular imaging parameters to estimate 3-, 5-, and 7-year overall survival [28]. Although such models were developed across broad prostate cancer populations and have not yet been validated specifically for focal therapy decision-making, they illustrate how quantitative molecular imaging may eventually complement conventional clinical, pathological, and MRI-based selection criteria.

Micro-ultrasound (microUS), with a frequency of 29 MHz and an imaging resolution of approximately 70 microns, reveals the ductal anatomy of the prostate in high detail and is non inferior to MRI in detecting clinically significant prostate cancer [29]. Through real-time lesion localization during FT, this approach facilitates direct placement of ablation needles into the tumor and enables immediate delineation of treatment boundaries [30]. Its main advantages over MRI are low cost, no need for contrast agents, quick accessibility, and real-time needle tracking capability that prevents recording errors in fusion software. Additionally, micro-US provides effective risk classification for patient selection in FT by standardizing tissue characterization through the PRI-MUS scoring system [31].

The utilization of artificial intelligence (AI)-empowered instruments in patient selection and ablation planning for FT can facilitate a degree of precision that exceeds the capabilities of conventional radiological interpretation in delineating tumor boundaries and volume. FDA-cleared software such as Unfold AI (Avenda Health) creates patient-specific 3D cancer maps by integrating T2-weighted axial MRI sequences, serum PSA levels, and biopsy findings (Gleason Score, cancer core length, and 3D localization) using a multimodal approach. By analyzing the prostate at the voxel level, these algorithms significantly reduce the tendency of standard MRI to underestimate tumor size (with an AI correlation of R^2^ = 0.76 compared to a traditional MRI-ROI correlation of R^2^ = 0.33) and more accurately identify flattened, nonspherical tumor geometries across prostate zones [32]. Furthermore, models such as MicroSegNet, which enables automatic segmentation in micro-ultrasound images, and ProstNFound, which generates a real-time heatmap of cancer suspicion, demonstrate that artificial intelligence is no longer limited to MRI but has become a critical guide for determining biopsy and treatment margins within the multimodal imaging ecosystem [29]. Beyond tumor segmentation and ablation planning, artificial intelligence may also contribute to focal therapy selection through prognostic and predictive modeling. Recent systematic evidence in urologic cancers suggests that digital pathology-based and multimodal AI models may improve risk stratification and outcome prediction, including survival estimation and identification of patients who may benefit from treatment intensification or de-escalation [33]. For focal therapy, such approaches are conceptually important because patient selection depends not only on lesion visibility and anatomical accessibility but also on the biological likelihood of progression, occult multifocal disease, and future need for salvage therapy. However, AI tools have not yet been prospectively validated as stand-alone decision-making instruments for focal therapy eligibility; therefore, their role should currently be considered adjunctive and hypothesis-generating.

Taken together, these emerging imaging and computational tools should be interpreted with appropriate caution. mpMRI remains the cornerstone of focal therapy planning, while PSMA PET/CT and micro-ultrasound should currently be considered complementary rather than substitutive modalities. Although PSMA PET/CT may improve local staging and detect MRI-occult clinically significant disease, focal therapy-specific thresholds, standardized interpretation criteria, and prospective evidence demonstrating improved long-term oncological outcomes remain limited. Similarly, micro-ultrasound offers real-time visualization and may improve targeting, but its integration into routine focal therapy pathways requires further validation. AI-assisted imaging and planning tools are also promising for tumor segmentation, margin planning, and risk prediction; however, they have not yet been prospectively validated as stand-alone instruments for treatment eligibility, ablation planning, or follow-up decisions.

## 4. Patient Selection—Patient Factors

Age alone is not a decisive criterion in patient selection for FT; instead, the patient’s overall health status and life expectancy play a central role [13]. International consensus statements emphasize that FT offers oncological benefit only in patients with a life expectancy of at least 10 years. In those with a shorter life expectancy, FT should be avoided, as treatment-related morbidity may outweigh any potential cancer control benefits and adversely affect the QoL [12].

### Age-Specific Considerations: Young and Elderly Patients

Young Patients: The primary goal of FT in young men is to decrease the rate of complications such as urinary incontinence and erectile dysfunction, which may result from radical methods such as surgery or radiation [2,6]. In younger patients, widespread screening programs often lead to early-stage detection, allowing FT to offer a means of ‘buying time’ while avoiding the morbidity associated with radical treatments. However, long-term oncological outcomes beyond 10–15 years remain unproven, and in the event of treatment failure, subsequent salvage surgery may be technically more challenging [34].

Elderly Patients: In accordance with the FALCON (FocAL therapy CONsensus) project and major international guidelines, the clinical narrative for FT is shifting away from chronological age toward individualized life expectancy as the primary determinant for candidacy. Rather than using age as a cutoff, international consensus mandates that patients should have a life expectancy of at least 10 years to ensure an oncological survival benefit from the intervention [12]. Especially for those showing disease progression while on active surveillance (AS), FT is considered an effective option to control the disease without requiring removal of the entire gland, provided their health status and expected longevity remain robust enough to warrant active treatment rather than a transition to watchful waiting [13].

## 5. Energy Selection

In FT, the tumor location is a pivotal factor in selecting the energy source to be utilized. This is due to the fact that no single energy type is universally ideal for all regions, and the treatment selection is based on an “à la carte” (customizable/selective) model tailored to the tumor’s anatomical features (Table 1) [35]. It is important to recognize that focal therapy modalities do not have equivalent levels of clinical maturity or evidentiary support. HIFU and cryotherapy have the longest clinical experience and the largest body of published oncological and functional outcome data, including use in both primary and selected salvage settings. IRE has gained increasing interest, particularly for lesions close to critical structures because of its non-thermal mechanism, but long-term comparative data remain limited. TULSA, focal laser ablation, and photodynamic therapy are promising technologies with specific technical advantages; however, their use remains more selective, and the available evidence is generally less mature or more dependent on specialized expertise and carefully selected cohorts. Therefore, energy selection should not be based solely on lesion location, but also on the maturity of evidence, operator experience, institutional infrastructure, and patient-specific oncological and functional priorities.

### 5.1. Posterior Lesions

Lesions in the posterior region of the prostate are generally the most suitable for HIFU [36]. Since HIFU energy is delivered via a transrectal probe, it allows for the precise and non-invasive targeting of lesions closest to the rectal wall. However, prostate calcifications pose a significant technical and oncological limitation for ultrasound-based focal therapies such as HIFU. These hardened structures act as a physical barrier that prevents ultrasound waves from effectively penetrating the tissue and may hinder the elimination of tumor cells [7,9]. Focal Laser Ablation (FLA) can also be applied transrectally for these lesions [36]. IRE is considered a safe, non-thermal option for diseases of the posterior region, provided that a 5 mm safety margin is left around the rectum [12,36].

### 5.2. Anterior Lesions

Anteriorly located prostate tumors are more amenable to transperineal needle-based modalities, including cryotherapy and IRE, owing to improved physical accessibility [36]. HIFU is significantly less effective for anterior lesions because the ultrasound waves must pass through many tissue planes before reaching the tumor from the probe, causing energy loss and attenuation. In large prostates (>40–50 mL), tumors in the anterior region may fall completely outside the effective focal range of the transrectal probe [9,37]. TULSA is more effective than HIFU for anterior diseases because it delivers energy “from the inside out” through the urethra; however, calcifications around the tumor or periprostatic calcifications may reduce treatment efficacy [38].

### 5.3. Apex Lesions and Critical Structures

The prostate apex represents the most challenging area to treat due to its proximity to the external urinary sphincter and the nerve fibers responsible for erection [36,39]. Preserving these structures is vital for maintaining the patient’s quality of life regarding urinary and sexual health:

- External Urinary Sphincter (Striated Sphincter): Located at the prostate apex, this structure is responsible for urinary continence. Damage to the sphincter, particularly from thermal energy sources, is a major risk factor for permanent urinary incontinence.
- Neurovascular Bundles (NVBs): These bundles contain the pro-erectile nerve fibers and are located posterolaterally to the prostate. Preservation of at least one NVB is crucial for maintaining spontaneous erections. The concentration of these fibers is particularly high near the posterior apex, making apically located tumors high-risk for sexual dysfunction.

Other critical structures are as follows:

- Membranous Urethra: This is the segment of the urethra that passes through the pelvic floor. Damage to the prostatic or membranous urethra can lead to complications such as urethral sloughing, strictures, or acute urinary retention. Certain modalities, like TULSA or cryotherapy, use cooling or warming devices to protect the urethral lining during ablation.
- Rectal Wall: Due to its close posterior proximity to the prostate, the rectal wall is at risk of thermal or mechanical injury. The most feared complication is the development of a recto-urethral fistula, which, while rare in primary FT (0–1%), requires careful management and safety margins of at least 5 mm [7].
- Bladder Neck: Situated at the base of the prostate, protecting the bladder neck helps preserve urinary control and prevents bladder neck contractures.
- Pubic Symphysis: This bone structure is located anteriorly to the prostate. It must be identified during planning to avoid mechanical injury during needle-based procedures (cryotherapy, IRE) or to prevent ultrasound waves from being blocked or absorbed by the bone, which can cause indirect heat damage.

#### 5.3.1. Thermal Risk

Traditional thermal energies such as cryotherapy and HIFU carry the risk of causing permanent urinary incontinence or erectile dysfunction. Therefore, when using thermal sources, it is recommended to maintain a safety margin of at least 5–10 mm from the sphincter [12,36].

#### 5.3.2. Non-Thermal Advantage (IRE)

IRE is the preferred method for lesions close to the apex and the neurovascular bundles. Since it uses electrical pulses instead of heat, it causes cell death while preserving the structural integrity of nerves, vessels, and sphincters [12,36].

#### 5.3.3. Urethral Protection

TULSA can protect the urethra using a cooled probe; cryotherapy requires a urethral warming catheter to prevent tissue sloughing during the procedure [38,40].

## 6. Balancing Oncological Control and Quality of Life

The primary goal of FT is to maintain the patient’s QoL while controlling cancer. Compared to radical treatments, FT offers significantly better functional outcomes; urinary continence rates are between 90 and 100%, while the success rate in preserving sexual function is markedly higher than with radical surgery [6]. Prospective studies have shown clinically significant recurrence-free survival rates of approximately 86% at 12 months and 81% at 24 months in intermediate-risk patients [41]. These recurrences are distinctively classified by their anatomical location: in-field recurrences, occurring in approximately 8.5% to 17% of cases, are typically ascribed to technical limitations such as targeting imprecision, inadequate energy delivery, or insufficient safety margins. Conversely, out-of-field recurrences, reported at similar rates (8.0% to 12.3%), are frequently categorized as “selection failures” arising from the multifocal biology of prostate cancer and the presence of MRI-invisible or undetected satellite lesions missed during initial sampling [7,41,42]. Notably, nearly half of all post-FT recurrences manifest outside the primary treatment zone [10]. One advantage of recurrences following FT is that the treatment can be repeated. Studies show that a second focal session (re-do FT) results in only “minor” adverse effects on urinary and erectile function [41,43]. Therefore, FT is ideal for patients who prefer to preserve their sexual and urinary functions over the complete elimination of oncological risk (radical treatment), but who are not suitable for AS due to the risk of cancer progression [2].

However, these oncological outcomes should be interpreted in the context of the current evidence base, which is still dominated by single-arm prospective cohorts, retrospective series, and registry-based data. Direct randomized comparisons between focal therapy and radical prostatectomy or radiotherapy are lacking, and available follow-up remains relatively short for a disease in which metastasis-free survival, cancer-specific survival, and overall survival often require long-term observation. Therefore, favorable short- and intermediate-term functional outcomes should not be interpreted as proof of oncological equivalence to standard whole-gland treatments.

The volume of tissue ablated in prostate cancer treatment (focal, partial/zonal, or whole gland) is the most fundamental factor directly affecting the patient’s QoL and functional outcomes after the procedure. The literature and expert opinions confirm that functional preservation increases as the treated area decreases, and the risk of side effects decreases as radical treatments are avoided.

### 6.1. Urinary Function

Pre-existing lower urinary tract symptoms (LUTS) are not an absolute contraindication for FT. While international consensus documents generally define ideal FT candidates as patients with mild-to-moderate voiding symptoms who are sexually active, the presence of significant LUTS alone does not preclude the use of FT [12,13]. However, since the effects of FT on current urinary symptoms have not yet been clearly established, it is recommended that patients, especially those with severe symptoms, be informed in detail and realistically before treatment [9,39].

Urinary symptoms and prostate volume are decisive factors in selecting the FT modality to be applied. In patients with prostate volume > 50 mL and significant LUTS who are candidates for HIFU, tissue-reducing procedures such as TURP or HoLEP are recommended before or concurrently with FT for both symptom control and technical success [44,45]. The TULSA modality offers a significant functional preservation advantage due to its active urethral cooling mechanism, which protects the urethra from thermal damage during the procedure [46].

The most important risk factors for urinary incontinence after FT are the tumor location and the thermal spread potential of the energy source used. Apical lesions, in particular, constitute the highest-risk area due to their proximity to the external urinary sphincter [12,36].

### 6.2. Sexual Function

Baseline erectile dysfunction does not constitute an absolute contraindication to FT [15]; however, sexually active patients aiming to preserve functional outcomes represent the ideal candidates [12]. Therefore, documenting baseline functions using validated scales such as the IIEF-5 is essential for managing patient expectations and objectively evaluating post-treatment outcomes [6,7].

It has been reported that FT has higher rates of erectile function preservation compared to radical surgery [7,41]. Targeted ablation of the index lesion allows for the preservation of at least one neurovascular bundle, thereby ensuring the continuation of spontaneous erections [6,24]. Meta-analysis data show that the rate of erectile function preservation at 12 months was 81.3% after focal ablation, compared to 61.7% after total gland ablation [6]. Additionally, it has been reported that the decline in IIEF scores after FT is limited and that the overall QoL is largely preserved [2].

The relationship between the anatomical location of the tumor and the priority of preserving erectile function is decisive in the choice of energy source. In posteriorly located lesions close to neurovascular bundles, methods such as IRE, which act through non-thermal mechanisms, are preferred due to the preservation of nerve structures [35]. Apical lesions, on the other hand, constitute the most high-risk area for sexual dysfunction due to the density of pro-erectile nerve fibers and proximity to the sphincter; in these cases, modalities that preserve neurovascular structures are recommended instead of thermal methods [47].

## 7. Focal Therapy in the Post-Radiotherapy Salvage Setting

Although the main focus of this review is primary focal therapy for localized prostate cancer, focal salvage therapy after definitive radiotherapy represents an important and clinically distinct indication. In patients with biopsy-proven intraprostatic recurrence after radiotherapy and no evidence of nodal or distant metastasis, local salvage therapy may provide disease control while delaying or avoiding androgen deprivation therapy. This is particularly relevant because systemic therapy remains a common treatment approach for radiorecurrent disease, despite its metabolic, cardiovascular, sexual, and quality-of-life consequences.

Recent evidence suggests that local salvage approaches can achieve meaningful intermediate-term disease control in selected patients. In a systematic review and meta-analysis including 31 studies and 4525 patients with locally recurrent prostate cancer after radiotherapy, Miszczyk et al. reported pooled 2-year and 5-year androgen deprivation therapy-free survival rates of 76.8% and 55.2%, respectively, and pooled 2-year and 5-year metastasis-free survival rates of 90.4% and 75.2%, respectively [48]. Severe or worse adverse event rates varied by modality, with relatively manageable rates reported for salvage HIFU and cryotherapy [48]. These data suggest that local salvage therapy alone may be a reasonable option for carefully selected patients who wish to defer systemic therapy.

However, salvage focal therapy differs substantially from primary focal therapy. Prior irradiation alters tissue vascularity, healing capacity, and baseline urinary function, thereby increasing the importance of careful toxicity assessment. Ideal candidates should have biopsy-confirmed local recurrence, concordant imaging findings on mpMRI and/or PSMA PET/CT, absence of nodal or distant metastases, acceptable baseline urinary function, and a lesion that can be treated with adequate safety margins. Focal salvage therapy may be most appropriate for unifocal or clearly localized recurrences, whereas multifocal, MRI-invisible, or anatomically extensive recurrence may require whole-gland salvage approaches or systemic treatment. Therefore, salvage focal therapy should be performed in experienced centers within structured follow-up protocols, with explicit counseling regarding recurrence risk, potential need for subsequent therapy, and higher complication risk compared with primary focal therapy.

## 8. Consensus Statements and Guidelines

### 8.1. FALCON Consensus

The FALCON (Focal Therapy Consensus) project is a current consensus developed with the participation of a broad and diverse group of physicians from over 30 countries to eliminate uncertainties and standardize focal therapy (FT) applications (Table 2) [4,12].

### 8.2. Strengths and Limitations of the FALCON Consensus

The FALCON consensus provides a useful contemporary framework for standardizing focal therapy practice, particularly in areas where prospective comparative evidence remains limited. Its main clinical contribution is the convergence of expert opinion around several practical principles: focal therapy should be considered primarily in carefully selected patients with organ-confined, clinically significant but limited-volume disease; high-quality mpMRI and combined targeted plus systematic biopsy are essential; Grade Group 2 disease represents the most appropriate candidate category; and Grade Group 3 disease requires more cautious counseling and intensified follow-up. However, FALCON should be interpreted as consensus-based guidance rather than high-level evidence. The Delphi methodology, the predominance of urologists among participants, and the absence of long-term randomized comparative data limit the strength of its recommendations. Moreover, as further consensus initiatives and prospective datasets emerge, these statements should be viewed as evolving rather than definitive. In this review, we therefore use FALCON to contextualize current practice and highlight areas of agreement, while emphasizing that unresolved issues such as the role of PSMA PET/CT, management of bilateral disease, cribriform or intraductal histology, and acceptable PSA thresholds require further validation.

### 8.3. Guidelines

International guidelines (EAU, AUA, NCCN) generally take a cautious and reserved stance toward FT due to the lack of long-term randomized data. The consensus view of these guidelines is that FT cannot yet be considered a “standard of care.” Given the preference-sensitive and technically nuanced nature of focal therapy, candidate selection should ideally occur within a multidisciplinary framework that includes urologists, radiation oncologists, radiologists or nuclear medicine physicians, pathologists, and, when appropriate, medical oncologists. Radiation oncology input is particularly relevant when counseling patients about focal therapy versus radiotherapy, evaluating prior-radiotherapy recurrence, and discussing salvage treatment options within the broader continuum of localized prostate cancer care. All guidelines agree that the patient must have a life expectancy of at least 8–10 years for oncological benefit to be achieved. AS remains the primary recommendation for low-risk (Grade Group 1) patients; however, the presence of aggressive variants such as cribriform pattern or intraductal carcinoma is considered a negative feature for FT or AS by both the EAU and AUA [49,50,51]. A summary of international guideline recommendations on focal therapy for prostate cancer is presented in Table 3.

**Figure 1:**
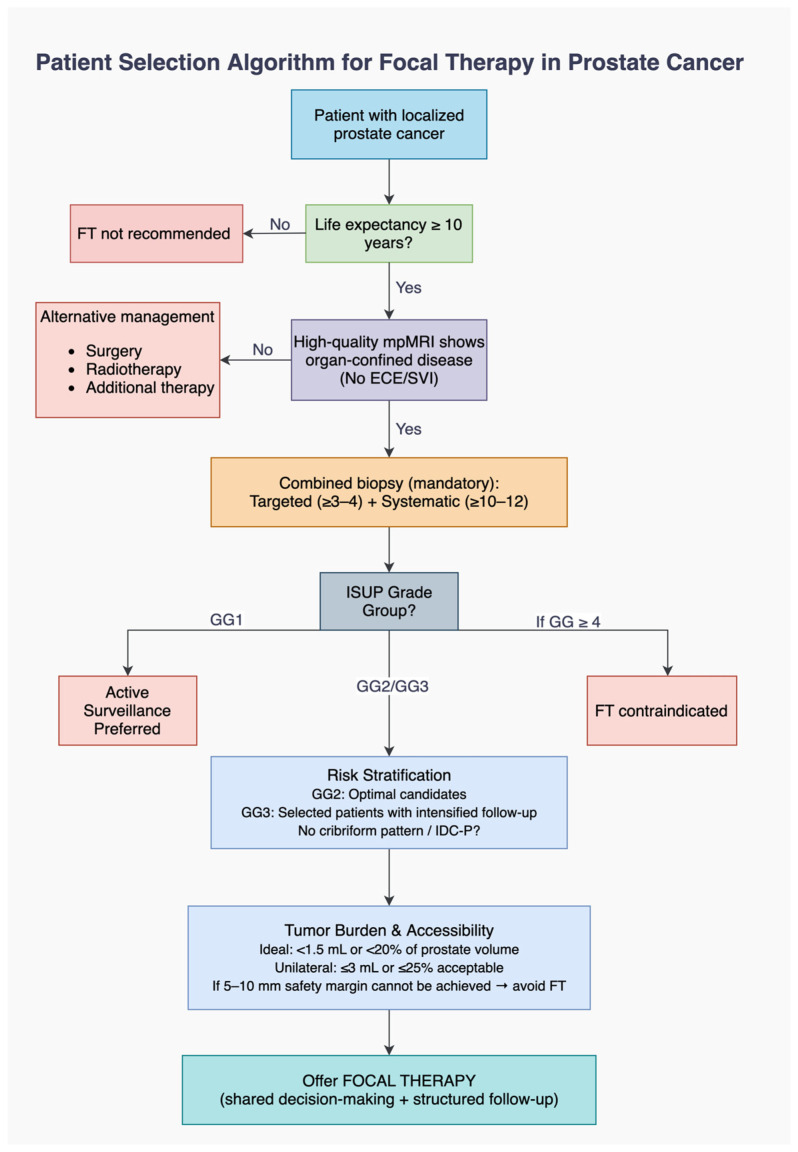
Patient selection algorithm for focal therapy in localized prostate cancer. This flowchart illustrates a stepwise, clinician-oriented decision-making process for selecting candidates for focal therapy. The algorithm integrates life expectancy, high-quality multiparametric MRI–based local staging, mandatory combined targeted and systematic biopsy, ISUP grade group stratification, and histopathological risk features such as cribriform pattern or intraductal carcinoma of the prostate. Tumor burden, laterality, and anatomical accessibility with adequate safety margins are subsequently assessed to determine technical feasibility. Patients meeting all criteria may be offered focal therapy within a shared decision-making framework and structured follow-up, whereas those who do not are directed toward active surveillance or radical treatment options.

Based on current evidence and consensus recommendations, a practical, clinician-oriented algorithm for patient selection in FT is illustrated in Figure 1.

## 9. Conclusions

In conclusion, patient selection for FT is a multidimensional process that requires a comprehensive evaluation of oncologic characteristics, histopathological risk profile, patient’s life expectancy, functional priorities, and data obtained from advanced imaging methods. Current evidence and consensus reports indicate that FT can provide acceptable oncological control while preserving QoL, particularly in selected intermediate-risk, organ-confined patients. However, ongoing uncertainties in areas such as multifocality, MRI-invisible disease, and aggressive histopathological subtypes remain unanswered. While new technologies such as PSMA PET, micro-ultrasound, and artificial intelligence-assisted imaging approaches are expected to further refine patient selection in the future, prospective studies conducted with standardized protocols and long-term oncological outcomes are still needed to clarify the place of FT in clinical practice.

## Figures and Tables

**Table 1:** Summary of Modality Selection by Location.

Tumor Location	Preferred Energy Source(s)	Primary Rationale
Posterior	HIFU	Direct transrectal access; well-documented safety.
Anterior	Cryotherapy, IRE, TULSA	Better transperineal or transurethral reach.
Apical	IRE, Brachytherapy	Spares nerves and sphincter via non-thermal mechanisms.
Peri-urethral	TULSA	Transurethral cooling protects the urethral lining.

HIFU, High-Intensity Focused Ultrasound; IRE, Irreversible Electroporation; TULSA, Transurethral Ultrasound Ablation.

**Table 2:** Key Patient Selection Criteria According to the FALCON Consensus.

Selection Criteria	Recommendation
**Life Expectancy and Age**	Chronological age alone should not be a decisive factor in patient selection. However, a minimum life expectancy of ≥10 years is mandatory for focal therapy to provide meaningful oncological benefit.
**Histopathological Criteria (ISUP Grade Groups)**	
ISUP Grade Group 1 (Gleason 3 + 3)	Focal therapy is not recommended in patients suitable for active surveillance.
ISUP Grade Group 2 (Gleason 3 + 4)	Considered the optimal candidates for focal therapy. In the presence of a cribriform pattern, focal therapy is preferred over active surveillance.
ISUP Grade Group 3 (Gleason 4 + 3)	Focal therapy may be offered in selected cases, but with caution due to increased oncological risk.
Contraindications	Patients with ISUP Grade Group > 3 (Gleason 8–10) and those harboring BRCA gene mutations are not suitable candidates for focal therapy.
**Imaging and Biopsy Requirements**	
mpMRI	mpMRI is mandatory. Focal therapy should not be offered when MRI is unavailable, of poor quality, or negative despite positive biopsy findings. Local staging should rely on MRI rather than digital rectal examination.
Biopsy Protocol	A combined approach including at least 3–4 targeted biopsies and 10–12 systematic biopsies is required before treatment.
PSMA PET/CT	PSMA PET/CT is not accepted as a substitute for mpMRI in patient selection.
**Anatomical Factors**	Tumor location alone is not a limitation, provided it can be safely accessed with the selected energy modality. Lesions located within 5 mm of the external urinary sphincter are generally not recommended for treatment.
**PSA Parameters**	PSA alone should not be used as a selection criterion. In patients with PSA > 15 ng/mL, a PSA density < 0.2 ng/mL/cm^3^ is emphasized as a requirement.

ISUP, International Society of Urological Pathology; PSA, prostate-specific antigen; PSMA PET/CT, prostate-specific membrane antigen positron emission tomography; mpMRI, multiparametric magnetic resonance imaging.

**Table 3:** Summary of International Guideline Recommendations on Focal Therapy for Prostate Cancer.

Guideline	Primary Position on Focal Therapy	Eligible Patient Groups	Key Remarks/Limitations
EAU	Focal therapy should be offered only within clinical trials or well-designed prospective registries	Selected low- and intermediate-risk patients	Not recommended for high-risk localized disease; current imaging modalities are considered insufficient to reliably detect all high-risk intraprostatic lesions
AUA	Not considered a standard treatment option due to lack of comparative high-quality evidence	Selected, well-informed low- and intermediate-risk patients; salvage setting after radiation failure	Patients must be informed of the investigational nature; HIFU is FDA-approved for tissue ablation but not specifically for prostate cancer; salvage cryotherapy or HIFU may be offered after biopsy-proven recurrence
NCCN	Classified as an emerging, non-standard treatment modality	Intermediate-risk: Should be utilized only in the context of a clinical trial. Low-risk: Discouraged as Active Surveillance (AS) is preferred. High-risk: Discouraged outside of trials.	Not recommended as routine primary therapy; in radiation-recurrent, non-metastatic disease, cryotherapy and HIFU are listed as category 2A options, and IRE as category 2B

EAU, European Association of Urology; AUA, American Urological Association; NCCN, National Comprehensive Cancer Network; HIFU, high-intensity focused ultrasound; IRE, irreversible electroporation.

## Data Availability

No original datasets were generated or analyzed during the preparation of this review. All data discussed in this article are derived from previously published studies, which are cited in the reference list.
